# Comparative Gene Expression Analyses Identify Luminal and Basal Subtypes of Canine Invasive Urothelial Carcinoma That Mimic Patterns in Human Invasive Bladder Cancer

**DOI:** 10.1371/journal.pone.0136688

**Published:** 2015-09-09

**Authors:** Deepika Dhawan, Melissa Paoloni, Shweta Shukradas, Dipanwita Roy Choudhury, Bruce A. Craig, José A. Ramos-Vara, Noah Hahn, Patty L. Bonney, Chand Khanna, Deborah W. Knapp

**Affiliations:** 1 Department of Veterinary Clinical Sciences, Purdue University College of Veterinary Medicine, Purdue University, West Lafayette, Indiana, United States of America; 2 Purdue Oncological Sciences Center, Purdue University, West Lafayette, Indiana, United States of America; 3 CCR Comparative Oncology Program, Center for Cancer Research, National Cancer Institute, Bethesda, Maryland, United States of America; 4 Department of Bioinformatics, Strand Genomics Inc, San Francisco, California, United States of America; 5 Department of Statistics, Purdue University, West Lafayette, Indiana, United States of America; 6 Department of Comparative Pathobiology, Purdue University College of Veterinary Medicine, Purdue University, West Lafayette, Indiana, United States of America; 7 Department of Oncology and Urology, Johns Hopkins University School of Medicine, Baltimore, Maryland, United States of America; 8 Purdue University Center for Cancer Research, Purdue University, West Lafayette, Indiana, United States of America; INRS, CANADA

## Abstract

More than 160,000 people are expected to die from invasive urothelial carcinoma (iUC) this year worldwide. Research in relevant animal models is essential to improving iUC management. Naturally-occurring canine iUC closely resembles human iUC in histopathology, metastatic behavior, and treatment response, and could provide a relevant model for human iUC. The molecular characterization of canine iUC, however, has been limited. Work was conducted to compare gene expression array results between tissue samples from iUC and normal bladder in dogs, with comparison to similar expression array data from human iUC and normal bladder in the literature. Considerable similarities between enrichment patterns of genes in canine and human iUC were observed. These included patterns mirroring basal and luminal subtypes initially observed in human breast cancer and more recently noted in human iUC. Canine iUC samples also exhibited enrichment for genes involved in *P53* pathways, as has been reported in human iUC. This is particularly relevant as drugs targeting these genes/pathways in other cancers could be repurposed to treat iUC, with dogs providing a model to optimize therapy. As part of the validation of the results and proof of principal for evaluating individualized targeted therapy, the overexpression of EGFR in canine bladder iUC was confirmed. The similarities in gene expression patterns between dogs and humans add considerably to the value of naturally-occurring canine iUC as a relevant and much needed animal model for human iUC. Furthermore, the finding of expression patterns that cross different pathologically-defined cancers could allow studies of dogs with iUC to help optimize cancer management across multiple cancer types. The work is also expected to lead to a better understanding of the biological importance of the gene expression patterns, and the potential application of the cross-species comparisons approach to other cancer types as well.

## Introduction

More than 70,000 people are expected to be diagnosed with bladder cancer in the United States in 2015, and 16,000 people are expected to die from the disease [1, 2]. High grade, muscle invasive urothelial carcinoma (iUC, also known as transitional cell carcinoma) is responsible for most bladder cancer related deaths. The five year survival rate for patients with muscle-invasive urinary bladder cancer is 69% if the cancer is still organ confined, 34% if lymph node metastases are present, and a mere 6% if the cancer has metastasized to distant organs. [2] Better management of iUC is urgently needed.

Further characterization of iUC at the genomic level is expected to lead to better management of iUC at several points including: early detection, prevention, prognostication, and treatment. The Cancer Genome Atlas (TCGA) project recently documented whole-exome sequencing of 131 muscle-invasive, high grade human bladder tumors and matched normal samples. Findings included novel as well as previously reported mutations and targets for treatment pertinent to human urothelial carcinoma. [3] Furthermore, unique to human iUC, i.e., not yet reported in other cancers, was the identified alteration of chromatin regulatory genes. This added knowledge opens up potential new avenues for treatment, thereby facilitating better management of the disease in the near future.

It has been well established that bladder cancer has two defined pathways leading to either superficial low grade bladder tumors or higher grade invasive bladder cancer, and that 50% of invasive bladder tumors harbor mutations/alterations in *P53* or *P53* pathways.[4, 5] However, emerging reports have highlighted two distinct gene expression profiles even within human iUC which curiously resemble patterns of the basal and luminal subtypes as characterized in human breast cancer. [3, 6–8] In addition, iUC has also been documented to display distinct subtypes enriched for *P53* pathways. [6, 7] As these new findings are applied in research to improve the outlook for people with iUC, relevant animal models are crucial.

Invasive urothelial carcinoma develops naturally in dogs. [9, 10] The disease resembles human iUC remarkably in its frequency, histopathology, heterogeneity, sites of metastases, and response to conventional chemotherapy. [9, 10] Although experimentally-induced rodent models are key in bladder tumor research, pet dogs with iUC are thought to offer a complementary highly-relevant model of invasive bladder cancer. The relevance of canine bladder cancer to human iUC includes substantial inter and intra-tumoral heterogeneity, the development of the cancer in subjects with intact immune system and bodily processes, and the aggressive invasive and metastatic behavior of the cancer. Clinical trials conducted in pet dogs with iUC could yield critical information to assist in the design of clinical trials in humans. [9] One example is the application of cyclooxygenase (COX) inhibitors in iUC where the beneficial effects were first documented in pet dogs with iUC and then similar biological changes observed in humans receiving COX inhibitors. [9–11] Additional trials ongoing in dogs with iUC, including targeted therapies and demethylating agents, could be of high translational value. [10, 12–14] With the compressed life span, dogs also provide a unique opportunity to study prevention strategies. [10] Although canine iUC appears to offer tremendous value as a translational model, to better use this model, it is important to understand the molecular characteristics and how those are similar to or different from human iUC.

This study was undertaken to define the gene expression patterns in canine iUC and to compare and contrast these findings with human iUC. Understanding the genomic similarities is expected to lead to new strategies to improve detection of iUC, intervene earlier in the course of disease, predict individual patient outcome, and more effectively manage the cancer overall.

## Methods

### Overview

**Fig 1:**
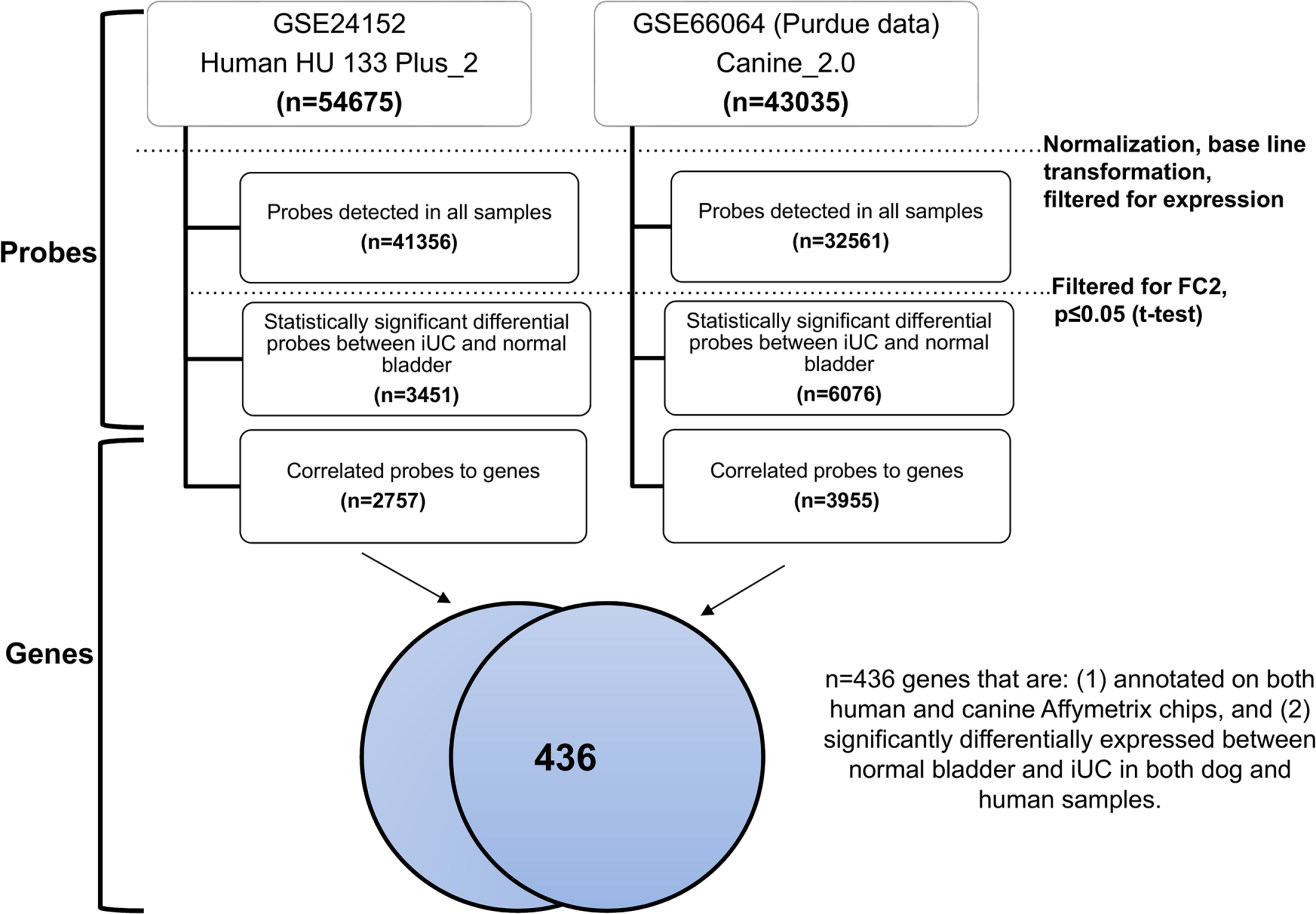
Flow chart and summary of cross-species analyses. Step-wise depiction of sample processing and cross-species analyses that were performed.

**Fig 2:**
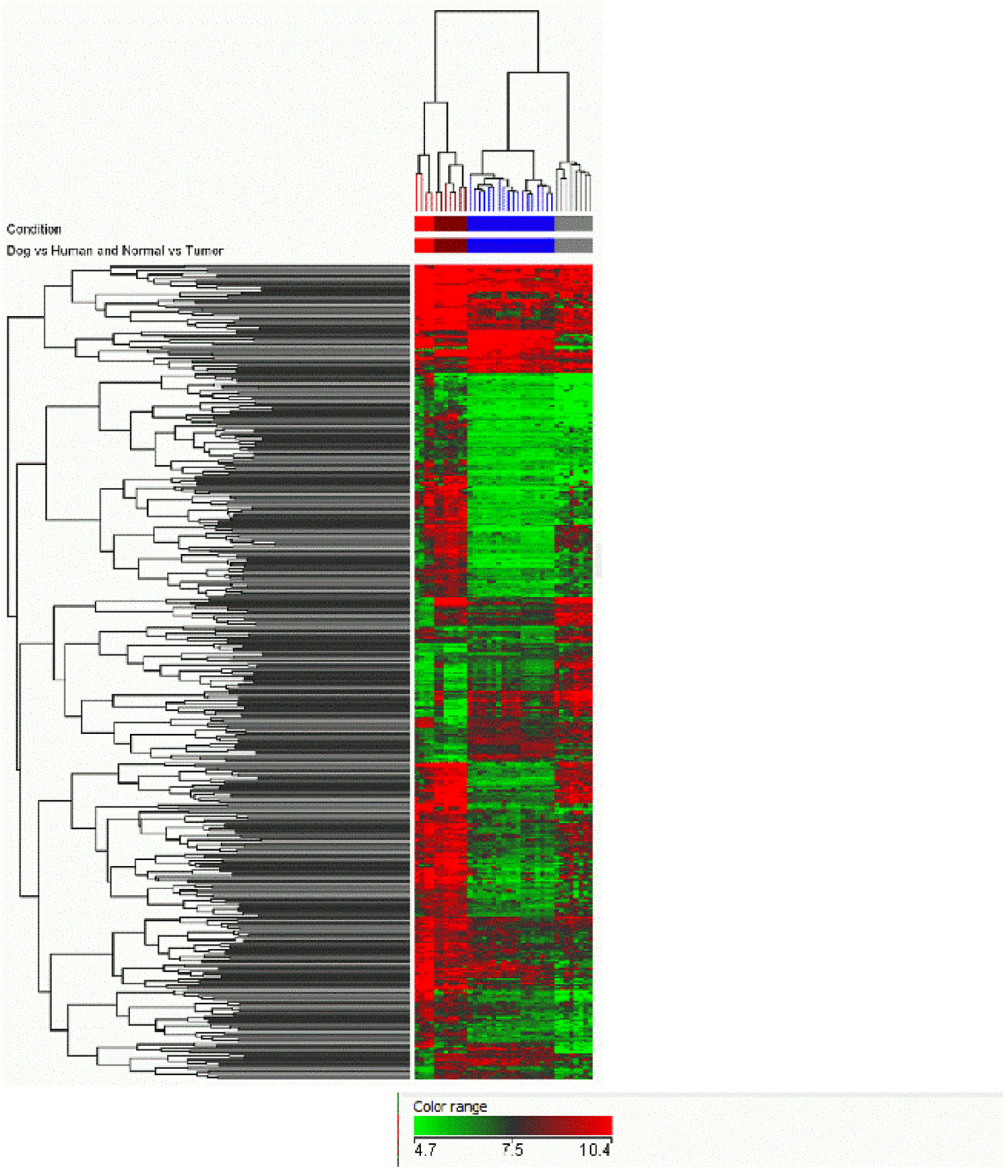
Canine and human iUC samples cluster together. A list of genes that are commonly annotated and significantly expressed (between normal and iUC, p<0.05, FC2) in dogs and humans, was generated. Hierarchical clustering was performed on these genes (n = 436) using Euclidean distance metrix. Figure illustrates that canine and human normal controls cluster together and these cluster separately from canine and human iUC samples. The iUC samples from dogs and humans clustered together. The color codes are: (1) red bar denoting canine normal bladder, (2) brown bar denoting normal human bladder, (3) blue bar denoting canine iUC samples, and (4) grey bar denoting human iUC samples.

**Fig 3:**
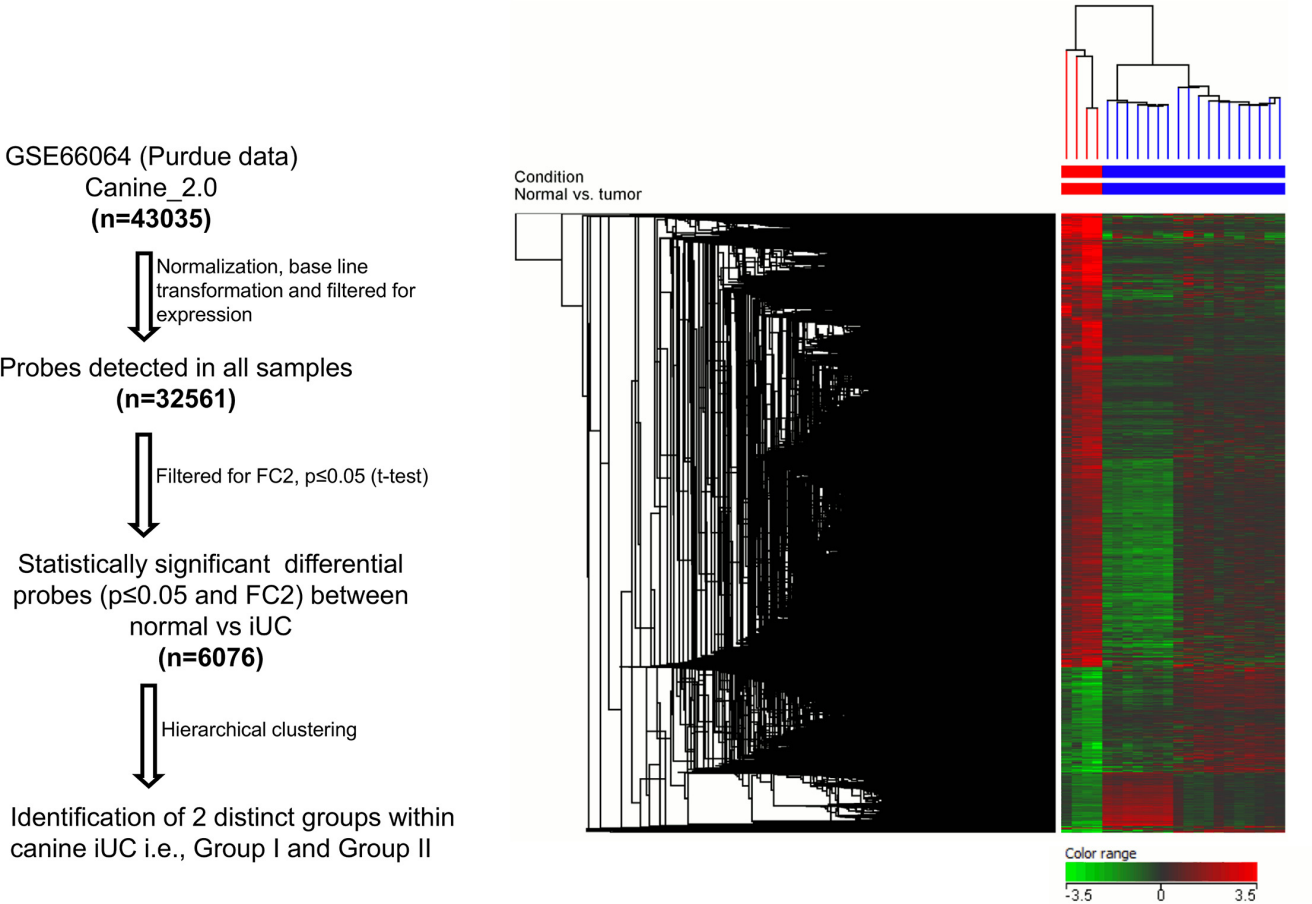
Canine iUC samples cluster as two distinct groups. Hierarchical clustering illustrates the differential expression of genes (p<0.05, FC2) between canine iUC samples vs. normal canine bladder. Furthermore, the canine iUC samples clustered in two distinct groups.

The Purdue University Animal Care and Use Committee and the owners of the pet dog approved collection of tissues from dogs with naturally-occurring invasive bladder cancer. The Purdue University Animal Care and Use Committee also approved the study reported in this manuscript. The samples were collected during routine veterinary care procedures that were needed to diagnose the naturally-occurring disease in the dog. The work included (1) microarray expression profile analysis to determine genes differentially expressed between canine iUC and normal canine bladder (Fig 1), (2) comparison of statistically significantly differentially expressed genes (differentially expressed between normal and iUC) enriched in both canine and human tumor samples (Fig 1), and (3) validation of over expression of EGFR protein in canine iUC samples and normal bladder specimens.

### Microarray Expression Profiling Analysis of Canine Samples

Gene expression profiling was performed using the Genome Array 2.0 (Affymetrix canine 2.0 with 18,000 *C*. *familiaris* mRNA/EST-based transcripts and over 20,000 non-redundant predicted genes, Affymetrix, Santa Clara, CA). Labeling, hybridization, and scanning of the microarray were performed at the Center for Cancer Research, Comparative Oncology Program, National Cancer Institute, Bethesda, MD. Total RNA was extracted from freshly isolated normal canine bladder from non-tumor bearing, healthy dogs (n = 4) and iUC samples prior to therapy (n = 18) using Trizol (Invitrogen, Carlsbad, CA) and purified using RNAeasy (Qiagen, Valencia, CA). Fresh tumor samples were collected by cystoscopy, and diagnosis confirmed by histopathology to be high grade iUC. Biotinylated amplified RNA was hybridized to the Affymetrix canine 2.0 chips. Four biological replicates were analyzed for the normal group (age matched non-tumor bearing dogs), and 18 biological replicates were analyzed for the iUC group. Statistical analyses of the expression patterns were performed using GeneSpring GX 12.6.1 (Agilent Technologies, Santa Clara, CA). Probes between 20–100 percentile were filtered to identify expression. Moderated t-test with Benjamini FDR multiple testing correction was used to generate a list of statistically significant probes. A filter of 2-fold change was used to identify up- or down- regulated probes. Differential expression analyses were done on genes that correlated to the probes. The criterion used for significantly dysregulated genes was a p value of ≤ 0.05 and a 2-fold or higher change. Hierarchical clustering was performed using Ward’s method to compute linkage and Euclidean distance metrics. The gene list was further analyzed for functional and pathway analysis using GeneSpring GX 12.6.1 (Agilent Technologies, Santa Clara, CA).

### Microarray Expression Profiling Analysis of Human Samples

Human microarray expression data were mined from the GEO database (GSE24152) which was run on the Affymetrix GeneChip Human Genome U133 Plus 2.0 Array platform. [15] GSE24152 was selected because of similar technology (Affymetrix platform) and inclusion of normal human bladder and treatment naive human iUC samples. This platform contains a complete coverage of the Human Genome U133 Set plus 6,500 additional genes for analysis of over 47,000 transcripts. The dataset included microarray expression data from human normal bladder (n = 7) and iUC samples (n = 8). In silico validation of this dataset was conducted against larger datasets GSE31684 and GSE5287 (95.6% overlap) to further confirm the agreement of the selected dataset against larger ones reported. The raw data (GSE24152) were processed and analyzed as described for canine samples using GeneSpring GX 12.6.1 (Agilent Technologies, Santa Clara, CA).

### Data Processing and Analyses

The data from each species were processed and analyzed independently. Human WikiPathways (Wikipathways.org) source was used for finding enriched pathways using the hypergeometric distribution for the p value calculation of individual significant pathway. The NCBI Homologene table was used to obtain the orthologous genes between dog and human for pathway mapping.

Human normal bladder and iUC data submitted to GEO database (ref: GSE24152) were used to mine the data of human differential expression in iUC as compared to normal bladder. [15] As conducted by the authors, bU28 and bU29 were excluded from the analyses.

A list of basal and luminal genes characterized previously in human breast cancer and implicated to be of importance in human invasive bladder cancer was developed. [3, 7, 8] This list of genes was used to perform unsupervised hierarchical clustering using Euclidean distance metrics on the same genes differently expressed between normal and canine iUC samples. GSEA analyses were conducted using GeneSpring GX 13.0 with datasets imported from the Broad Institute using 5 minimum number of genes with a 100 permutations and a q cut off value of 0.3. KEGG Pathway analyses were conducted using DAVID.

### Immunohistochemistry

Immunohistochemistry of paraffin-embedded tissue sections was performed using manufacturer’s instructions (BioCare, Concord, CA) with modifications. [13] Briefly, histopathologically confirmed canine normal bladder and iUC slides were deparaffinized and hydrated, followed by antigen retrieval using proteinase K (Dako Corporation, Carpinteria, CA). The slides were incubated with EGFR mouse monoclonal antibody clone 528 (Pierce, Rockford, IL) overnight at 4°C. The slides were incubated with mouse probe (BioCare Medical, Concord, CA) followed by MACH 4. The signal was detected using 3,3'-diaminobenzidine reagent (DAKO Corporation, Carpinteria, CA); slides were counterstained with hematoxylin (Sigma, St Louis, MO), dehydrated, and mounted in permount (Sigma, St Louis, MO). Paired control slides were stained using Universal Negative control serum (BioCare Medical, Concord, CA). Normal canine skin served as a positive control for specificity of immunostaining. The percent of positively immunostained tumor cells was categorized as 0 to 3 as follows: 0 = <10% of cells, 1 = 10–19% of cells, 2 = 20–49% of cells, and 3 >50% of cells. The intensity of immunostaining was graded on a scale of 0–3 where 0 = no staining, 1 = equivocal staining, 2 = moderate to intense staining, and 3 = highest intensity staining. The IHC score was determined by multiplying the category for percent positive cells by the staining intensity such that 9 was most intense and 2 was the least. An IHC score of 0–1 was considered negative.

## Results

### Cross Species Analyses of Gene Expression Between Canine and Human Normal Bladder and iUC Samples

Cross-species analyses were performed using a two-step process (Fig 1). First, the commonly annotated genes on both human and canine chips were identified, and second was the identification of differentially expressed genes between normal bladder and iUC both in canine and human samples (FC2, t-test unpaired p<0.05). This step-wise process identified 436 genes which were subjected to unsupervised hierarchical clustering, and the results are illustrated in Fig 2. The list of genes is available in Table A in S1 File. The characteristics of the dogs providing iUC samples are summarized in Table B in S1 File. Regions signifying concordance and differences in expression data, i.e., upregulated or downregulated genes, between canine and human iUC samples can be visualized in Fig 2.

### Hierarchical Clustering of Canine iUC Samples

**Fig 4:**
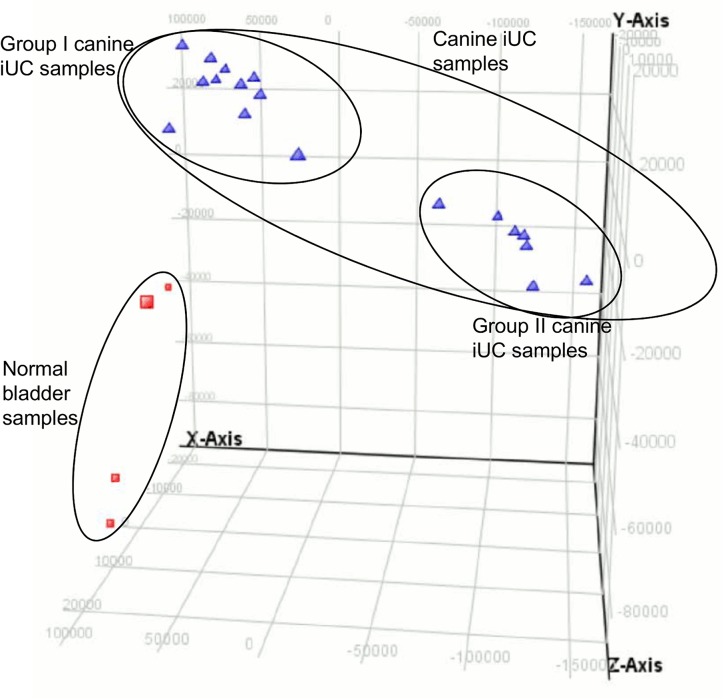
Principal Component Analyses (PCA) plot of normal canine bladder and iUC samples. A PCA plot was generated using normalized data. The PCA plot shows clear separation between normal samples and canine iUC samples. In addition, the PCA plot also demonstrates clear segregation of the canine iUC samples into two groups i.e., group I and group II.

On performing unsupervised hierarchical clustering, the canine iUC samples clustered as two distinct groups (Fig 3). The clustering of groups was independent of sex, age, breed, cancer grade, stage, or clinical outcome. As visualized in the PCA plot, not only did the normal bladder samples cluster separately from the tumor samples, but 11 tumor samples (designated as Group I) segregated separately from the other 7 tumor samples (designated as Group II) (Fig 4). A total of 2012 pathways were found to be enriched in canine iUC samples. Utilizing DAVID to analyze the KEGG pathways enriched while analyzing canine iUC samples indicated the erbb signaling pathway to be enriched significantly (Table C in S1 File). Gene ontology (GO) analyses of canine iUC samples indicated aberrations in biological processes (87%), molecular functions (9%) and cellular components (4%) (Table D in S1 File). Gene Set Enrichment Analyses (GSEA) revealed enrichment of genes in the categories of hallmark, C3 and C4 gene sets (Table E in S1 File).

**Fig 5:**
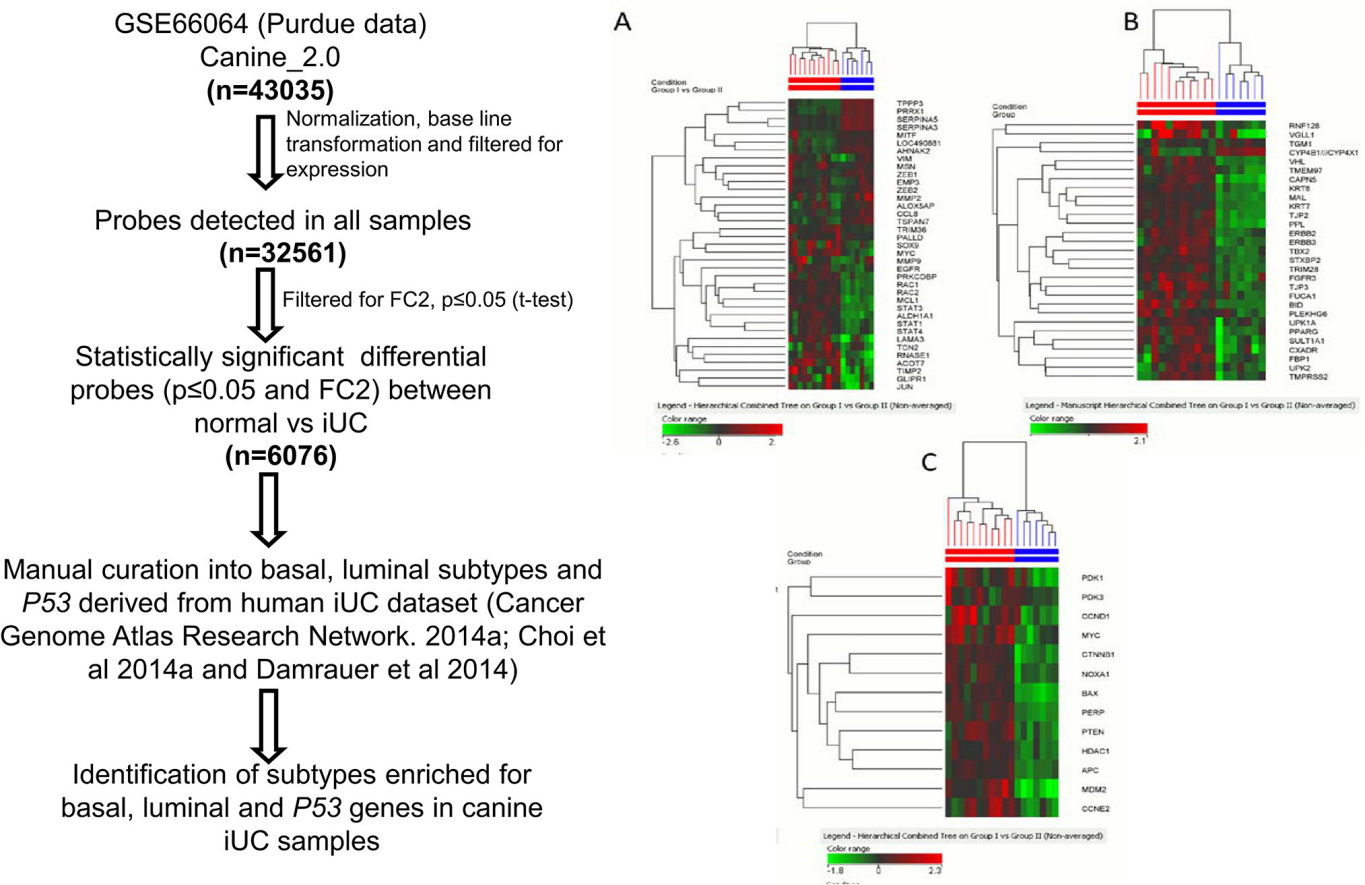
Analyses of canine iUC samples reveal enrichment for basal and luminal subtypes and also for genes in *P53* pathways. A list of genes representing basal and luminal subtypes and those involved in *P53* pathways was manually generated from published human iUC dataset (Cancer Genome Atlas Research Network. 2014a; Choi et al 2014a and Damrauer et al 2014). A second list was generated, using these genes as a reference, which were also significantly expressed in canine iUC samples and hierarchical clustering was performed using Euclidean distance metrix. Heat maps indicate genes enriched for basal (A) and luminal (B) subtypes of breast cancer in the canine iUC samples and also indicate enriched genes in the *P53* pathways (C). Red and blue bars above the heat map indicate the clean separation of samples into the previously observed groups. There was no clear segregation of basal and luminal genes.

Some of the genes representing basal and luminal subtypes of human breast cancer and *P53* pathways associated genes that were also differentially expressed between canine normal bladder and iUC were subjected to hierarchical clustering using all of the dog samples (Table F in S1 File). Upon clustering, canine iUC samples were found to be enriched for basal (Fig 5A) and luminal (Fig 5B) subtypes identified in human bladder cancer (resembling the breast cancer subtypes) and also showed patterns of enrichment of *P53* pathways associated genes (Fig 5C).

### Enriched Epidermal Growth Factor–Epidermal Growth Factor Receptor (EGF-EGFR) Pathway in Canine iUC Samples

**Fig 6:**
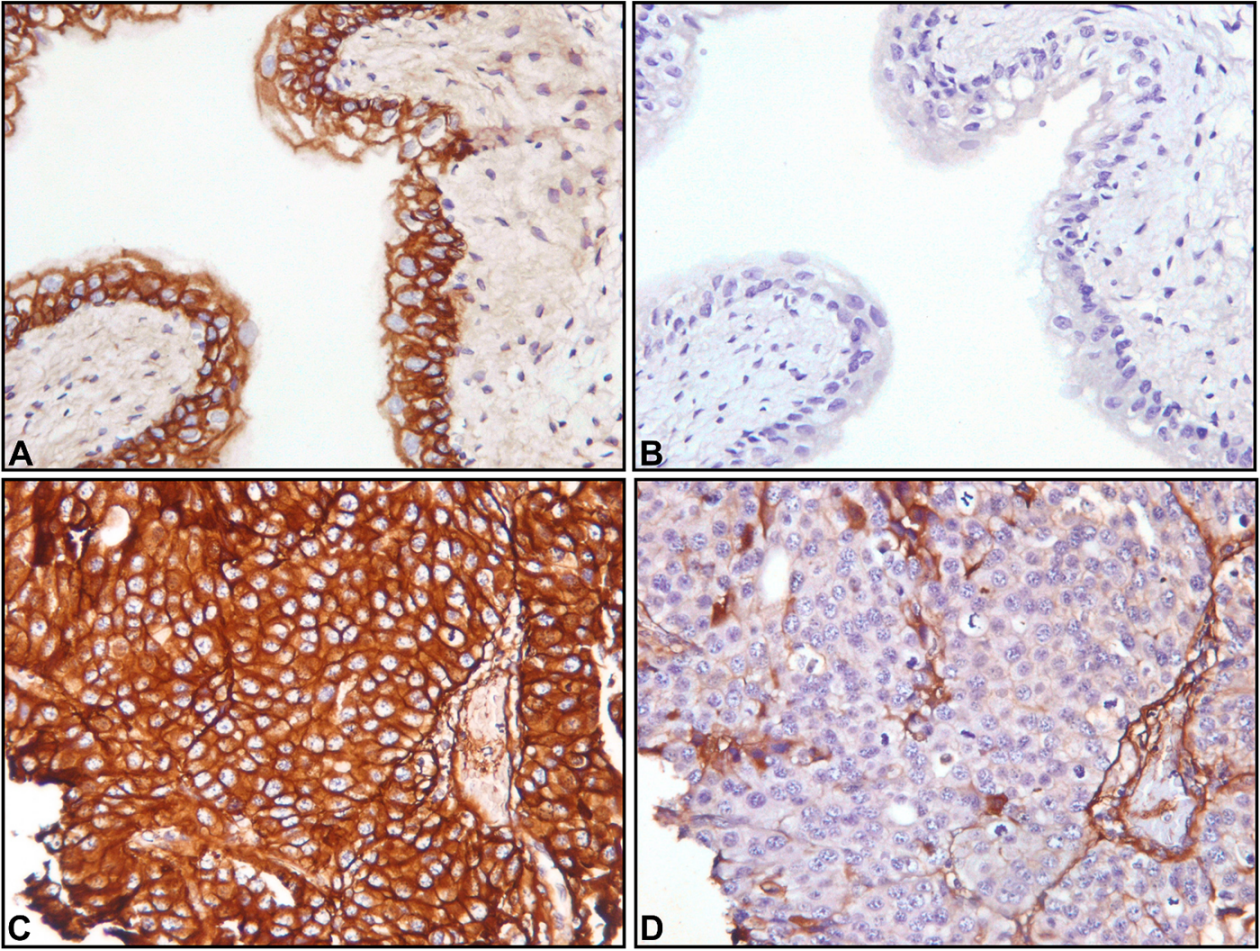
Immunohistochemical detection of EGFR expression in normal and iUC canine tissues. Photomicrographs of canine normal bladder (A and B) and canine iUC samples (C and D) demonstrating immunoreactivity to EGFR. Paired negative controls were used for each specimen (B and D). Please note membrane immunostaining of tumor cells (C) and normal urothelium (A).

The EGF-EGFR pathway has received considerable attention in human iUC, and trials of therapies aimed at this pathway are underway in people with iUC. [16, 17] EGFR expression has also recently been reported in canine iUC. [18] The *EGFR* pathway was found to be enriched in canine iUC with a p<4.65E-05. To further confirm EGFR expression, immunohistochemistry was performed on tissue samples from 48 dogs with histopathologically confirmed intermediate to high grade iUC. The median age of the dogs was 11.5 years (range 6–17.5 years), and multiple breeds were represented. The tumor samples were from 1 intact female, 1 intact male, 26 spayed female and 20 neutered male dogs. EGFR expression was detected in 35 of 48 (73%) primary tumors (Fig 6). Immunoreactivity was noted in the epithelial cells in 8 of 8 normal bladders from dogs (≥ 90% cells positive, 2–3+ staining intensity; IHC score of 9).

## Discussion

Approximately 16,000 people are expected to die from iUC in the United States in 2015. [2] Cystectomy is considered the standard frontline treatment for people with apparent organ-confined cancer, but approximately half of these patients harbor metastases that emerge after surgery. [19] Although platinum based chemotherapy can control metastases initially, the development of resistance to the agents is common, and there is not a well-defined effective second line treatment for metastatic iUC. [19, 20] It is essential to develop better means to reduce the risk of iUC, to detect it earlier when it could be more effectively treated, and to provide better care for advanced cancer. Fortunately, the increasing understanding of aberrant pathways involved in the etiology and progression of bladder cancer is expected to aid in the development of personalized, targeted, and more effective therapy options. [3]

Treating cancer is no longer limited to treating a pathologic type. A shift is taking place towards treating the molecular signature or subtype thereby leading to more individualized treatment. This subtyping crosses “organ-specific” cancers as well as “species” as clearly validated by the data presented herein making dogs a biologically relevant model for iUC. Lessons learned in other organ-specific cancers involving similar molecular signatures can be harnessed and applied in a tailored manner. [4, 21–23]

As new management strategies are being developed, the availability of a highly relevant animal model, e.g. dogs with iUC, could greatly facilitate progress in the field. [9, 10] In particular, the recent promise of the administration of immune checkpoint inhibitors in metastatic human bladder cancer patients has heightened the appreciation of relevant immunocompetent animal models to optimize therapy. [24, 25] Rodents with experimentally-induced bladder tumors are the mainstay in pre-clinical bladder cancer research, but these models can fail to mimic the invasive and metastatic behavior, the molecular pathways involved, and the inter- and intra-tumoral heterogeneity that are hallmarks of human iUC. Naturally-occurring iUC in dogs offers several features that complement other animal models of bladder cancer. [10] The dogs, which are pet dogs, have functional immune systems, and share the environment, water, and often food with the people they live with. There is marked heterogeneity in canine iUC, as is the case in humans. Dogs are large enough for serial samples of blood, urine, and tumor tissue (collected via cystoscopy) to be collected over time. In addition, the compressed life span of dogs offers opportunities to test prevention strategies and therapeutic options in a shortened window of time compared to humans. Treatment options tested in dogs with naturally occurring iUC could potentially offer insights and better treatment options not only for humans with iUC but for humans with other cancers which have been genotyped to have genetic aberrations similar to those reported in canine iUC. One such example of the comparative value of clinical trials conducted in dogs [9, 26] is the beneficial biological changes observed following the administration of a selective *COX-2* inhibitor in the neo-adjuvant setting in humans. [11]

The findings from the work presented here provide additional compelling evidence for dogs serving as relevant and important models to improve the outlook for people with iUC, and other cancers as well. Several of the enriched pathways reported herein, i.e., focal adhesion, EGF-EGFR, signaling by ERBB2, angiogenesis etc. have been documented to be of importance not only in human iUC, but in several other human cancers. [3, 27–35] Of particular excitement is the enrichment of genes such as *UPK3A*, *ERBB2*, *FOXA1*, *ZEB1*, *ZEB2*, *CDH1*, *VIM*, *S100A1*, *S100A9*, *EGFR* and *PPARG* that have been highlighted recently to be of importance in human iUC. [3, 7, 8, 36]

The enthusiasm of finding similarities and of the validation of targets and pathways in canine iUC when comparing to the human disease, is coupled with an acknowledgment that differences will exist. The difference in the expression of certain probes is highlighted, and easy to visualize when clustering human and dog normal samples and iUC samples. This is not unexpected or discouraging, as these differences could add to the understanding of the molecular interactions that drive the development and progression of iUC. Another difference recently reported between iUC in dogs and humans is the presence of a *BRAF* mutation (V600E) in canine iUC that is rare in human iUC. [3, 37] Alterations in MAPK signaling occur in iUC in both species, but a *BRAF* mutation is more commonly identified in canine iUC. Even with the presence of the *BRAF* mutation in canine iUC, similarities do exist in the expression patterns across the two species.

One example of potential value in translational research is the EGF-EGFR signaling pathway in iUC. EGFR and EGF-EGFR signaling has been documented to be of critical relevance in iUC, and in other human cancers as well. [27–29, 32, 38, 39] Over expression of EGFR protein has been reported in 79% of human iUC (stage T3-T4) as detected by immunohistochemistry. [40] The finding that a comparable 73% of canine iUC tissues overexpress EGFR provides strong support for the comparative model and offers opportunities to further explore a target of recognized importance in human iUC. EGFR is being targeted for treatment in humans with iUC. [41–43] and other cancers. [38, 39, 44–46] An EGFR targeted therapy trial is underway in dogs at the Purdue University Veterinary Teaching Hospital (unpublished data, Aguilar, Knapp).

Another critical finding from the work was the identification of expression patterns similar to those observed in human bladder cancer, i.e. luminal and basal subtypes as validated in human breast cancer. [3, 6–8] Canine iUC samples were found to be enriched for these subtypes. For instance, some of the genes enriched in canine iUC samples i.e., *PPARG*, *TBX2*, *ERBB2*, *ERBB3* are genes indicative of luminal subtypes of breast cancer in humans. [3, 6, 47] Furthermore, examples of genes enriched in the canine iUC samples resembling the basal subtype of breast cancer, and implicated to be critical in human iUC, are *MMP9*, *EGFR*, *JUN* and *MYC*. In another example, the epithelial mesenchymal transition markers implicated to play an important role in human iUC i.e., *ZEB1*, *ZEB2*, *VIM*, *CDH1* and *CLDN3*, were also significantly enriched in canine iUC samples. Many of these genes/gene products have been targeted in other cancers. Mutations or aberrations in the *P53* pathways have been documented to play active roles in human iUC. [8] Genes such as *MDM2*, *CTNNB1*, *HDAC1*, *APC*, *BAX*, *PTEN* and *NOXA1* were enriched and differentially expressed among the canine iUC samples. Although GSEA [48] did not reveal basal and luminal subtypes when the canine data were analyzed by this method, this could have been due to small sample size. Understanding the similarities between human and canine iUC, and also similarities across other cancer types, e.g. breast cancer, is expected to offer newer modalities for treatment and also for predicting treatment outcome for patients with bladder cancer.

The results of the expression analyses of canine iUC provide further value for naturally-occurring invasive bladder cancer in pet dogs as a relevant model for human invasive bladder cancer. It is anticipated that this and other studies characterizing iUC in dogs will increase the incorporation of dogs with iUC in translational research. Although there are many compelling reasons to consider translational research involving dogs, there are challenges and limitations to this approach. [49] Much of the scientific community is not aware or knowledgeable of the existence and potential value of studies of dogs with naturally-occurring cancer. In addition, the criticism has been raised that there are not enough dogs for studies. Currently, single institution studies can enroll 50–75 dogs per year. Trials consortia such as the National Cancer Institute’s Comparative Oncology Trials Consortium, can enroll more cases than single institutions (https://ccrod.cancer.gov/confluence/display/CCRCOPWeb/Comparative+Oncology+Trials+Consortium). This consortium includes academic veterinary oncologists at 20 veterinary colleges across the United States and Canada. The authors acknowledge, however, that if the scientific community should need to have several dog studies performed simultaneously, then more veterinary scientists and institutions would have to engage in this type of research, and new strategies would have to be developed to identify and enroll more dogs. Even by conservative estimates, there are 20,000 dogs which develop naturally-occurring iUC yearly in the United States, but many of these dogs do not undergo diagnosis and treatment. Furthermore, it is obvious that in cancer research, dog studies are never going to replace rodent studies in which a group of animals which are very similar and which have more predictable cancer can be acquired and studied simultaneously at a planned period of time. It is much better to view dog studies as a complement to the research done in rodents. Dogs develop heterogenous cancer which has innate and acquired drug resistance, similar to the hallmarks of human cancer. While some view this as a weakness of the dog model, the authors would propose that this is actually a great opportunity to address important challenges facing human cancer patients. Another limitation to conducting dog studies is the financial cost. Dog studies typically cost much more than rodent studies, and granting agencies are not always willing to provide this level of funding for animal work. Dog studies, however, are far less expensive than human trials. If a carefully designed and conducted study in dogs with spontaneous cancer could mean the difference between success and failure of a multi-million dollar human study, then the cost of the dog study could be a very sound investment. And finally, it is important to know that many pet owners, as well as comparative oncology researchers, view clinical trials in dogs as a win-win-win scenario. The individual dog gains access to a treatment that is expected to be beneficial and well tolerated, and pet owners embrace the possibility that their dog’s participation will provide information that could help other dogs and humans facing cancer.

In conclusion, expression analyses of canine iUC has further validated naturally-occurring invasive bladder cancer in pet dogs as a relevant model for the human disease The findings documented herein are in line with those reported in the human literature. These include, but are not limited to, the clusters in canine iUC that are enriched for basal, luminal subtypes of human breast cancer, and genes involved in *P53* pathways. These findings also provide evidence for the view proposed by TCGA that some common pathways are involved in the development of multiple types of cancer despite different sites/organs of origin. [3]

## Supporting Information

S1 File Table A: List of genes that are commonly expressed in dogs and humans, and that are differentially expressed between normal and iUC (p<0.05). Table B: Characteristics (gender, age, grade, stage at diagnosis, and stage at death) of dogs providing iUC samples which were analyzed. Table C: List of KEGG pathways enriched in canine iUC samples analyzed. Table D: Gene Ontology distribution of canine iUC samples analyzed. Table E: List of genes enriched by GSEA analyses. Table F: List of genes used to perform hierarchical clustering to classify genes as basal or luminal subtypes of breast cancer and genes participating in *P53* pathways.
(PDF)
