# Supplementary material for: Comparative Gene Expression Analyses Identify Luminal and Basal Subtypes of Canine Invasive Urothelial Carcinoma That Mimic Patterns in Human Invasive Bladder Cancer
Source: PLoS One. 2015 Sep 9;10(9):e0136688. doi: 10.1371/journal.pone.0136688 (PMC4564191; doi:10.1371/journal.pone.0136688)
Supplement: S1 File — Table B: Characteristics (gender, age, grade, stage at diagnosis, and stage at death) of dogs providing iUC samples which were analyzed. Table C: List of KEGG pathways enriched in canine iUC samples analyzed. Table D: Gene Ontology distribution of canine iUC samples analyzed. Table E: List of genes enriched by GSEA analyses. Table F: List of genes used to perform hierarchical clustering to classify genes as basal or luminal subtypes of breast cancer and genes participating in P53 pathways. (PDF) [file pone.0136688.s001.pdf]

Table A. List of genes that are commonly expressed in dogs and humans, and that are differentially expressed between normal and iUC ($p<0.05$).

Gene Symbol	Entrez Gene dog
A2M	477699
ABCA8	490898
ABI3BP	478544
ACSS3	475414
ACTA2	477587
ACTC1	478250
ACTG2	475792
ACTR3	403702
ACVR2A	476140
ADCY5	403859
AGMAT	487430
AGR2	482333
AHI1	476212
AHR	475251
AIF1	474841
AKT3	442964
ALDH2	610941
AMICA1	610790
ANKRD13D	483700
ANKRD35	475825
ANXA6	479325
AP1S2	611468
ARHGAP24	478463
ARHGAP9	474413
ARHGEF10	609511
ASB2	490836
ATP10D	482134
ATP2A2	403878
ATP2A3	491437
ATP8A1	607976
AURKA	485940
BAG2	610506
BAI3	481870
BIN1	483870
BIRC5	442936
BMP5	474944
BMX	491750
BNC2	612979
BOC	487979
BOD1L1	479089
C1R	477707
C1S	486714
C7	489221
CASP4	403724
CBLB	487965
CBLC	612343
CBX5	477593
CCDC58	478583
CCDC91	486622
CCL14///CCL14-CCL15///CCL15	480603
CCL2	403981
CCNA2	483845
CCNB1	608420
CCNB2	478324
CCNL1	485730
CCNT2	476126
CD48	488642
CD59	475945

CDC37L1	476341
CDCA3	611617
CDH11	479696
CDO1	474637
CELF2	607310
CENPA	475692
CEP112	480462
CFD	485095
CFH	478952
CFI	478515
CFL2	490649
CFLAR	488471
CGN	483198
CHD9	478128
CHPT1	610214
CLIC4	487367
CLYBL	476974
CNN1	484937
CNRIP1	612567
CNST	611848
COL14A1	475085
COL15A1	474778
CORO1A	489949
COX7A1	612614
CPA3	485707
CPE	475492
CPEB4	479287
CPQ	477946
CPXM2	486927
CREM	403887
CRY1	474528
CSRP1	607054
CTSF	476010
CUTC	477793
DAB2	479353
DACH1	485489
DCN	403904
DDR2	478987
DENND5A	476844
DKK3	476857
DLGAP5	480331
DMD	606758
DMXL1	474631
DNAJB4	479982
DPT	490355
DPYSL3	487204
DTNA	490488
DYNC1H1	480437
DZIP1	476964
E2F1	485839
EBF1	479312
ECM2	476361
ECSCR	478026
EEF1A1///LOC100653236	403506
EFEMP1	474604
EHMT2	474851
EIF1	403674
EIF1B	477030
EMCN	609940
EMILIN1	475696
EMP1	486676
ENTPD6	485564
EPAS1///LOC100652809	474578

EPB41L2	484127
EPB41L3	480198
ERCC6L	491955
ETV4	403641
F13A1	478711
F2RL1	488940
FAM110A	609631
FAM129A	480041
FAM176C	478402
FAM190B	479268
FAM63B	487577
FAM65B	488247
FAM82A1	483037
FANCD2	484659
FASN	483378
FBLN1	474468
FBN1	478293
FBXL5	479085
FCER1A	478970
FGF13	492174
FGL2	475902
FHL1	492162
FIGF///PIR-FIGF	491749
FILIP1L	478537
FKBP7	488424
FLNA	481084
FLRT2///LOC100506718	490820
FNBP1	480695
FOLR2	476816
FOXM1	486743
FST	479336
FXYD1	476487
FXYD6	610831
GAS6	607745
GEM	611634
GHR	403721
GLIPR1	474452
GLT8D2	612239
GLUL	403443
GMFG	611578
GNAI1	478227
GNG2	610817
GOLGA2	480708
GPCPD1	485778
GPR65	490821
GPRASP1	480996
GRK5	486915
GSN	474819
GTSE1	608855
GUCY1A2	479455
GULP1	478834
HECTD2	486795
HSD11B1	449023
HSD17B10	480930
HSPB7	487423
HTR2B	403881
IFITM1///IFITM2	483397
IGF1	610255
IGSF10	477114
IL33	403810
IL6	403985
INADL	479550
IQGAP2	479177

JAM2	478392
JAZF1	475265
KANSL1L	478894
KCNJ8	486639
KCNMB1	403983
KIAA0101	610710
KIF20A	474693
KIF4A	491941
KIFC1	607916
KITLG	403507
KLC3	484448
KLF10	481992
KLHDC1	490677
KLHDC3	474907
KLHDC5	477659
KLHL5	612886
KPNA5	484094
KRT8	486513
LAMA4	475034
LGALS8	479193
LHFP	485998
LIMS2	612895
LIX1L	608332
LMO2	609006
LPGAT1	609933
LPHN2	490193
LPP	478670
LRRC49	487626
LSM12	480504
LSP1	611553
LTBP4	444849
LUM	482599
MAN1A1	476275
MAP1B	478092
MATN2	491431
MBNL1	477116
MCAM	489368
MCC	611208
MCM10	487128
MCM7	479737
MED21	477664
MEF2C	479155
MEOX1	607271
METTL7B	474390
MFAP4	489531
MFAP5	477701
MGLL	476511
MORF4L1	610223
MPDZ	474708
MRC1	487114
MRPL52	480240
MRTO4	487406
MYBL2	477232
MYH11	479836
MYL9	485856
MYLK	488012
NAA15	483817
NAV1	490238
NBEA	477305
NBR1	480509
NCAPG	479132
NCAPH	475741
NDC80	480190

NDFIP1	478044
NDN	488693
NEK2	480021
NFE2L2	478813
NFIA	479552
NFIB	474709
NMRK1	484162
NOVA1	490632
NPM3	477801
NPR2	474762
NR2F2	479026
NRN1	612757
NRP1	477955
NSMCE4A	477849
NUF2	478988
NUSAP1	475154
NUTF2	479681
OGN	610704
OLFML1	476839
OLFML3	483126
OSBPL1A	480761
P2RX1	491223
PALLD	477351
PAM	479145
PARP8	479339
PCDH7	488843
PCGF5	486794
PCM1	475618
PCSK6	488703
PDE4DIP	475817
PDE5A	403825
PDGFD	479460
PER1	489488
PEX3	484015
PGM5	476332
PGR	403621
PHKA2	480857
PHKB	478139
PIEZO2	490554
PIGR	474357
PIP4K2A	608414
PIP5K1B	476331
PJA2	479143
PKIG	610925
PLAGL1	484018
PLCL1	478858
PLK1	489971
PLOD2	485702
PMP22	479509
POC1A	484741
PPAP2A	607962
PPAT	611407
PPM1K	478473
PPP1R12A	475411
PPP3CB	479248
PRC1	488742
PRDM5	483850
PRKAR2B	475887
PRKCB	489968
PRKCZ	479577
PRKD1	609091
PROM1	488816
PROS1	478529

PRRC2C	480075
PSIP1	474712
PSPH	489783
PTGDS	403740
PTGS1	403544
PTPLB	608764
PTPMT1	483622
PTPRG	484706
PTTG1	479307
PVRL4	609931
PXK	476575
PYGO2	490440
RAB27A	608699
RAB30	476787
RAB40C	611450
RABEP1	489451
RABGAP1L	480064
RACGAP1	486549
RAD51	403568
RAP1A	483225
RASA3	476998
RASGRP2	612101
RASL12	487600
RASSF3	608312
RASSF6	482195
RASSF8	477666
RBPJ	479122
REV3L	481963
RFC5	477499
RGS18	607858
RHOD	611862
RNASE4	611048
RRM2	482963
RSPO3	476287
RUFY3	475168
RUNX1T1	487044
RWDD1	609552
SCD	486839
SCHIP1	478678
SEC14L1	483338
SELE	403999
SELENBP1	475847
SERINC1	476277
SERPING1	475966
SFRP2	475471
SGCE	475233
SGMS1	477582
SHE	612268
SIK3	479426
SKA3	609400
SLC15A2	488006
SLC25A39	490938
SLC27A4	491317
SLIT2	595148
SLMAP	476577
SMOC2	476270
SOX5	486635
SPARCL1	478470
SPATA6	475364
SPATS2L	478862
SPC24	611174
SPG20	477303
SPRY2	485504

SRPX	491828
SSBP2	479160
ST14	489278
ST7L	612836
STAT4	478845
STAT5B	490969
STMN1	478175
STOX2	482912
SUV39H1	491868
SVEP1	474799
SVIL	477965
SYTL2	485154
TACC1	475581
TAGLN	479424
TBC1D1	479115
TCEAL1	481000
TCF4	403949
TENC1	477599
TGFB1I1	489932
TGFBR3	490163
TLE1	476314
TLE4	476317
TMEM100	609661
TMEM176A	475535
TMEM176B	610914
TMEM63A	490389
TMEM88	607946
TMOD1	474771
TNC	481689
TOM1L1	491093
TOP2A	480525
TPX2	477186
TRAIP	608724
TRIP13	609426
TROAP	608844
TSC22D1	476927
TSPAN2	612755
TTLL7	479976
TUBA1A	608147
TYROBP	476477
UBE2C	485898
UBFD1	479806
UBQLN4	490419
USP15	474424
USP5	486718
USPL1	486019
UST	476238
VIM	477991
WBP4	476935
WFDC1	489682
WLS	611491
YAP1	479465
ZBTB16	489398
ZBTB20	487987
ZBTB38	477096
ZC3H13	485453
ZEB1	477966
ZEB2	483909
ZMAT1	480993
ZMYND8	477252
ZNF331	414288
DES	497091
SOD2	477299

Table B. Gender, age, grade, and stage at diagnosis and stage at death of canine iUC samples analyzed.

Group	Gender	Age at Dx (yrs)	Grade	Stage at Dx	Stage at death
**Group I**	FS	13	High	T2N0M0	T2N0M0
**Group I**	FS	14	High	T2N0M1	T2N0M1
**Group I**	FS	13	High	T2N0M0	T2N0M1
**Group I**	FS	10	High	T3N0M0	T3N1M1
**Group I**	MN	8	High	T2N0M0	T2N1M1
**Group I**	FS	9	High	T2N0M0	T3N0M0
**Group I**	FS	14	High	T2N0M0	T2N0M0
**Group I**	MN	9	High	T3N0M0	T3N1M1
**Group I**	MN	7	High	T2N0M0	T3N1M0
**Group I**	MN	9	High	T3N0M0	T3N1M1
**Group I**	FS	9	High	T2N0M0	T2N0M1
**Group II**	MN	10	High	T2N0M1	T2N0M0
**Group II**	FS	13	High	T2N0M0	T2N0M0
**Group II**	M	10	High	T2N0M0	Follow-up Not Available
**Group II**	FS	10	High	T3N0M0	T2N0M1
**Group II**	FS	13	High	T3N0M0	Follow-up Not Available
**Group II**	FS	13	High	T2N0M0	Follow-up Not Available
**Group II**	M	12	High	T2N0M0	Follow-up Not Available

FS: Female Spayed; MN: Male Neutered and M: Male

Table C. List of KEGG pathways enriched in canine iUC samples analyzed.

Category	Term	Count	%	P-Value	Benjamini
KEGG_PATHWAY	Focal adhesion	66	2	5.10E-07	9.50E-05
KEGG_PATHWAY	Tight junction	43	1.3	1.90E-04	1.80E-02
KEGG_PATHWAY	Endocytosis	54	1.6	1.20E-03	7.40E-02
KEGG_PATHWAY	Leukocyte transendothelial migration	36	1.1	2.90E-03	1.30E-01
KEGG_PATHWAY	Spliceosome	42	1.2	3.30E-03	1.20E-01
KEGG_PATHWAY	ECM-receptor interaction	26	0.8	4.70E-03	1.40E-01
KEGG_PATHWAY	Renal cell carcinoma	23	0.7	5.60E-03	1.40E-01
KEGG_PATHWAY	Pyrimidine metabolism	27	0.8	7.90E-03	1.70E-01
KEGG_PATHWAY	Basal transcription factors	14	0.4	1.00E-02	1.90E-01
KEGG_PATHWAY	Pathways in cancer	79	2.3	1.20E-02	2.00E-01
KEGG_PATHWAY	Arginine and proline metabolism	18	0.5	1.50E-02	2.20E-01
KEGG_PATHWAY	RNA polymerase	10	0.3	1.60E-02	2.20E-01
KEGG_PATHWAY	RNA degradation	19	0.6	2.10E-02	2.70E-01
KEGG_PATHWAY	ErbB signaling pathway	25	0.7	2.40E-02	2.80E-01
SP_PIR_KEYWORDS	phosphoprotein	58	1.7	2.50E-02	1.00E+00
SP_PIR_KEYWORDS	alternative splicing	13	0.4	2.60E-02	9.50E-01
KEGG_PATHWAY	Valine, leucine and isoleucine degradation	15	0.4	2.60E-02	2.90E-01
COG_ONTOLOGY	DNA replication, recombination, and repair / Transcription / Translation, ribosomal structure and biogenesis	5	0.1	3.10E-02	7.20E-01
GOTERM_BP_FAT	reproductive process in a multicellular organism	8	0.2	3.10E-02	1.00E+00
GOTERM_BP_FAT	multicellular organism reproduction	8	0.2	3.10E-02	1.00E+00
KEGG_PATHWAY	Purine metabolism	38	1.1	3.30E-02	3.30E-01
KEGG_PATHWAY	Small cell lung cancer	23	0.7	3.50E-02	3.20E-01
UP_SEQ_FEATURE	short sequence motif:Nuclear localization signal	6	0.2	3.60E-02	1.00E+00
KEGG_PATHWAY	Homologous recombination	10	0.3	3.70E-02	3.30E-01
SP_PIR_KEYWORDS	developmental protein	9	0.3	4.00E-02	9.60E-01
KEGG_PATHWAY	Hypertrophic cardiomyopathy (HCM)	23	0.7	4.00E-02	3.30E-01
GOTERM_CC_FAT	tight junction	5	0.1	4.10E-02	1.00E+00
GOTERM_CC_FAT	occluding junction	5	0.1	4.10E-02	1.00E+00
KEGG_PATHWAY	Pancreatic cancer	21	0.6	4.30E-02	3.40E-01
GOTERM_BP_FAT	mammary gland development	4	0.1	4.50E-02	1.00E+00
KEGG_PATHWAY	Cell adhesion molecules (CAMs)	32	0.9	4.50E-02	3.40E-01
SP_PIR_KEYWORDS	Tight junction	5	0.1	4.60E-02	9.30E-01
UP_SEQ_FEATURE	splice variant	12	0.4	4.60E-02	1.00E+00
KEGG_PATHWAY	Propanoate metabolism	11	0.3	4.80E-02	3.50E-01
KEGG_PATHWAY	Thyroid cancer	11	0.3	4.80E-02	3.50E-01
KEGG_PATHWAY	Adherens junction	21	0.6	5.00E-02	3.40E-01
SP_PIR_KEYWORDS	cytoplasm	27	0.8	5.00E-02	9.10E-01
KEGG_PATHWAY	Acute myeloid leukemia	17	0.5	5.10E-02	3.40E-01
GOTERM_BP_FAT	chemical homeostasis	13	0.4	5.30E-02	1.00E+00
GOTERM_BP_FAT	cell motion	9	0.3	5.40E-02	1.00E+00
GOTERM_BP_FAT	cell migration	8	0.2	5.50E-02	1.00E+00
GOTERM_BP_FAT	di-, tri-valent inorganic cation homeostasis	8	0.2	5.50E-02	1.00E+00
UP_SEQ_FEATURE	short sequence motif:Cell attachment site	4	0.1	6.20E-02	1.00E+00

SP_PIR_KEYWORDS	cell adhesion	7	0.2	6.30E-02	9.20E-01
KEGG_PATHWAY	Axon guidance	33	1	6.30E-02	3.90E-01
GOTERM_BP_FAT	homeostatic process	16	0.5	6.60E-02	1.00E+00
GOTERM_BP_FAT	vasculature development	9	0.3	6.70E-02	1.00E+00
GOTERM_CC_FAT	internal side of plasma membrane	8	0.2	7.10E-02	1.00E+00
KEGG_PATHWAY	Butanoate metabolism	10	0.3	7.40E-02	4.20E-01
INTERPRO	DEATH-like	4	0.1	7.80E-02	1.00E+00
GOTERM_BP_FAT	respiratory system development	4	0.1	7.80E-02	1.00E+00
GOTERM_BP_FAT	striated muscle tissue development	4	0.1	7.80E-02	1.00E+00
GOTERM_BP_FAT	Wnt receptor signaling pathway	4	0.1	7.80E-02	1.00E+00
GOTERM_BP_FAT	muscle tissue development	4	0.1	7.80E-02	1.00E+00
GOTERM_BP_FAT	respiratory tube development	4	0.1	7.80E-02	1.00E+00
GOTERM_BP_FAT	lung development	4	0.1	7.80E-02	1.00E+00
KEGG_PATHWAY	Regulation of actin cytoskeleton	48	1.4	8.00E-02	4.40E-01
KEGG_PATHWAY	Chemokine signaling pathway	41	1.2	8.00E-02	4.30E-01
COG_ONTOLOGY	Translation, ribosomal structure and biogenesis	20	0.6	8.40E-02	8.30E-01
GOTERM_MF_FAT	identical protein binding	8	0.2	8.40E-02	1.00E+00
GOTERM_BP_FAT	posttranscriptional regulation of gene expression	3	0.1	8.70E-02	1.00E+00
GOTERM_BP_FAT	cell motility	8	0.2	8.80E-02	1.00E+00
GOTERM_BP_FAT	localization of cell	8	0.2	8.80E-02	1.00E+00
KEGG_PATHWAY	Mismatch repair	8	0.2	9.20E-02	4.70E-01
GOTERM_CC_FAT	cell-cell junction	7	0.2	9.20E-02	1.00E+00
SP_PIR_KEYWORDS	wnt signaling pathway	3	0.1	9.30E-02	9.60E-01
KEGG_PATHWAY	Ubiquitin mediated proteolysis	32	0.9	9.50E-02	4.60E-01
KEGG_PATHWAY	Endometrial cancer	15	0.4	9.80E-02	4.70E-01

Table D. Gene Ontology distribution of canine iUC samples analyzed.

GO ACCESSION	GO Term	p-value	corrected p-value	Count in Selection	% Count in Selection	Count in Total	% Count in Total
GO:0005622	intracellular	2.63E-24	2.63E-19	2083	67.38919	8698	59.899456
GO:0044424	intracellular part	5.78E-24	2.89E-19	1975	63.89518	8174	56.29089
GO:0005515|GO:0045308	protein binding	7.24E-22	2.41E-17	1637	52.96021	6649	45.788857
GO:0043226	organelle	2.74E-21	6.84E-17	1749	56.58363	7175	49.411198
GO:0043229	intracellular organelle	2.18E-19	3.64E-15	1713	55.418957	7064	48.646786
GO:0043227	membrane-bounded organelle	2.13E-19	3.64E-15	1558	50.4044	6323	43.543835
GO:0043231	intracellular membrane-bounded organelle	2.73E-17	3.90E-13	1506	48.722095	6140	42.28359
GO:0071840|GO:0071841	cellular component organization or biogenesis	6.27E-16	7.84E-12	704	22.7758	2620	18.042835
GO:0005737	cytoplasm	2.10E-15	2.34E-11	1408	45.5516	5760	39.66669
GO:0016043|GO:0044235|GO:0071842	cellular component organization	9.30E-15	9.30E-11	683	22.096409	2559	17.622753
GO:0005488	binding	8.89E-13	8.08E-09	2255	72.953735	9887	68.0876
GO:0051270	regulation of cellular component movement	3.04E-12	2.54E-08	133	4.3028145	372	2.5618072
GO:0044085|GO:0071843	cellular component biogenesis	1.46E-11	1.12E-07	300	9.705597	1018	7.0105367
GO:0044237	cellular metabolic process	5.25E-11	3.75E-07	1236	39.98706	5086	35.025135
GO:0044444	cytoplasmic part	6.66E-11	4.44E-07	975	31.54319	3930	27.06425
GO:0032991	macromolecular complex	7.62E-11	4.76E-07	768	24.846329	3019	20.790579
GO:0040012	regulation of locomotion	1.01E-10	5.97E-07	125	4.0439987	357	2.4585083
GO:2000145	regulation of cell motility	1.28E-10	7.10E-07	117	3.7851827	331	2.2794573
GO:0022607|GO:0071844	cellular component assembly	4.20E-10	2.21E-06	275	8.896797	946	6.514703
GO:0030334	regulation of cell migration	5.27E-10	2.64E-06	111	3.591071	317	2.1830451
GO:0044422	organelle part	9.04E-10	4.30E-06	854	27.6286	3482	23.979065
GO:0044446	intracellular organelle part	1.17E-09	5.32E-06	830	26.852152	3373	23.228428
GO:0034330	cell junction organization	1.02E-08	4.44E-05	41	1.3264316	84	0.57847255
GO:0050839	cell adhesion molecule binding	1.34E-08	5.57E-05	39	1.2617276	79	0.54403967
GO:0043234	protein complex	2.63E-08	1.05E-04	641	20.737625	2539	17.485022
GO:0008152	metabolic process	3.88E-08	1.49E-04	1545	49.983826	6596	45.42387
GO:0044260|GO:0034960	cellular macromolecule metabolic process	4.90E-08	1.82E-04	869	28.113878	3537	24.357826
GO:0005634	nucleus	5.57E-08	1.99E-04	917	29.666775	3784	26.058811
GO:0005739	mitochondrion	5.83E-08	2.01E-04	325	10.514397	1188	8.181254
GO:0051128	regulation of cellular component organization	7.23E-08	2.41E-04	266	8.605629	960	6.611115
GO:0005623	cell	1.02E-07	3.18E-04	2273	73.53607	10128	69.74726
GO:0044464	cell part	1.02E-07	3.18E-04	2273	73.53607	10128	69.74726

GO:0071704	organic substance metabolic process	1.11E-07	3.35E-04	1313	42.47816	5549	38.213623
GO:0065010	extracellular membrane-bounded organelle	1.96E-07	5.59E-04	30	0.9705597	57	0.39253494
GO:0043230	extracellular organelle	1.96E-07	5.59E-04	30	0.9705597	57	0.39253494
GO:0034329	cell junction assembly	2.35E-07	6.36E-04	28	0.9058557	52	0.35810205
GO:0044238	primary metabolic process	2.31E-07	6.36E-04	1265	40.925266	5343	36.794987
GO:0043933|GO:0034600|GO:0034621	macromolecular complex subunit organization	2.73E-07	7.18E-04	233	7.5380135	818	5.6332207
GO:0043170|GO:0043283	macromolecule metabolic process	3.72E-07	9.53E-04	983	31.802006	4086	28.138557
GO:0070062	extracellular vesicular exosome	4.77E-07	0.001191407	29	0.9382077	56	0.38564837
GO:0045216	cell-cell junction organization	5.27E-07	0.001285098	34	1.0999676	73	0.5027202
GO:0061024|GO:0016044	membrane organization	6.36E-07	0.00151392	92	2.976383	279	1.9213552
GO:0006996	organelle organization	7.33E-07	0.001705573	390	12.617276	1504	10.357413
GO:0030336	negative regulation of cell migration	8.58E-07	0.001948932	43	1.3911356	101	0.69554436
GO:0051641	cellular localization	1.49E-06	0.003312104	272	8.799741	1022	7.0380826
GO:0030198	extracellular matrix organization	1.97E-06	0.004285208	52	1.6823034	132	0.9090283
GO:0044428	nuclear part	2.13E-06	0.004522508	364	11.776124	1405	9.675642
GO:0051271	negative regulation of cellular component movement	2.21E-06	0.004611854	44	1.4234875	107	0.73686385
GO:2000146	negative regulation of cell motility	2.30E-06	0.004691135	43	1.3911356	104	0.7162041
GO:0043062	extracellular structure organization	2.59E-06	0.005124325	52	1.6823034	133	0.9159149
GO:0040013	negative regulation of locomotion	2.61E-06	0.005124325	47	1.5205435	117	0.8057296
GO:0071822	protein complex subunit organization	2.90E-06	0.005576789	203	6.567454	709	4.8825836
GO:0032989	cellular component morphogenesis	4.16E-06	0.007846991	150	4.8527985	529	3.643
GO:0030155	regulation of cell adhesion	4.43E-06	0.008205314	70	2.2646394	209	1.4392948
GO:0051174	regulation of phosphorus metabolic process	5.38E-06	0.009777189	309	9.996765	1172	8.07107
GO:0007010	cytoskeleton organization	6.67E-06	0.011914755	150	4.8527985	514	3.5397012
GO:0030030	cell projection organization	7.01E-06	0.012291025	138	4.4645743	488	3.36065
GO:0007167	enzyme linked receptor protein signaling pathway	7.17E-06	0.012365893	103	3.332255	318	2.1899319
GO:0072657	protein localization to membrane	1.20E-05	0.02014125	45	1.4558395	116	0.798843
GO:0005178	integrin binding	1.21E-05	0.02014125	23	0.74409574	46	0.3167826

GO:0008092	cytoskeletal protein binding	1.27E-05	0.020763341	145	4.6910386	508	3.4983816
GO:0019220	regulation of phosphate metabolic process	1.38E-05	0.02230822	305	9.867357	1168	8.043523
GO:0005681	spliceosomal complex	1.45E-05	0.023054989	42	1.3587836	107	0.73686385
GO:0022603	regulation of anatomical structure morphogenesis	1.56E-05	0.0236129	130	4.2057586	438	3.0163212
GO:0051272	positive regulation of cellular component movement	1.54E-05	0.0236129	64	2.0705273	191	1.3153365
GO:0044267	cellular protein metabolic process	1.52E-05	0.0236129	545	17.631834	2193	15.102265
GO:0010647	positive regulation of cell communication	1.69E-05	0.025185391	186	6.01747	668	4.600234
GO:0033036	macromolecule localization	2.01E-05	0.029617546	243	7.8615336	941	6.48027
GO:0048583	regulation of response to stimulus	2.32E-05	0.031798415	450	14.558395	1807	12.444047
GO:0000166	nucleotide binding	2.31E-05	0.031798415	497	16.07894	2026	13.952208
GO:0040017	positive regulation of locomotion	2.29E-05	0.031798415	64	2.0705273	193	1.3291095
GO:1901265	nucleoside phosphate binding	2.31E-05	0.031798415	497	16.07894	2026	13.952208
GO:0022604	regulation of cell morphogenesis	2.28E-05	0.031798415	70	2.2646394	214	1.4737277
GO:1902580	single-organism cellular localization	2.51E-05	0.033529498	56	1.8117114	159	1.0949659
GO:1902578	single-organism localization	2.51E-05	0.033529498	56	1.8117114	159	1.0949659
GO:0044802	single-organism membrane organization	2.73E-05	0.035924397	78	2.5234551	247	1.7009848
GO:0030335	positive regulation of cell migration	3.02E-05	0.039280653	61	1.9734714	184	1.2671304
GO:0070372	regulation of ERK1 and ERK2 cascade	3.24E-05	0.041504692	39	1.2617276	97	0.6679981
GO:0048584	positive regulation of response to stimulus	3.60E-05	0.043426543	219	7.085086	825	5.681427
GO:0007044	cell-substrate junction assembly	3.50E-05	0.043426543	12	0.3882239	17	0.11707183
GO:0051649	establishment of localization in cell	3.60E-05	0.043426543	220	7.117438	825	5.681427
GO:0002685	regulation of leukocyte migration	3.48E-05	0.043426543	25	0.80879974	66	0.45451415
GO:0030031	cell projection assembly	3.57E-05	0.043426543	50	1.6175995	147	1.012327
GO:0031252	cell leading edge	4.04E-05	0.04804903	62	2.0058234	182	1.2533572
GO:0036094	small molecule binding	4.16E-05	0.04888807	531	17.178907	2182	15.026513

Molecular Function	8	9.41176471
Biological Process	74	87.0588235
Cellular Components	3	3.52941176
	85	

Table E. List of genes enriched by GSEA analyses.

#Gene Set : CCAWWNAAGG_V$SRF_Q4	#Gene Set : GNF2_CCNB2	#Gene Set : HALLMARK_UV_RESPONSE_DN	#Gene Set : V$SRF_C	#Gene Set : V$SRF_01	#Gene Set : V$SRF_Q4	#Gene Set : V$SRF_Q5_01	#Gene Set : V$SRF_Q6
AARSD1///BECN1	AURKA	ABCC1	ACTB	KCNMB1	ACTB	ACTB	ACTB
ACTA1	BIRC5	ACVR2A	ACTB///ACTBL2///ACTG1	MYL6	ACTB///ACTBL2///ACTG1	ACTB///ACTBL2///ACTG1	ACTB///ACTBL2///ACTG1
ACTA1///ACTC1	BUB1	ADD3	NR4A1	TPM1	CSDA	CSDA	COL1A1
ACTB	CCNA2	ADORA2B	MAPK14	LOC100686101///PLCB3///PRDX5	NR4A1	COL1A1	NR4A1
ACTB///ACTBL2///ACTG1	CCNB2	AGGF1	DMD	WDR1	EDN1	CDKN1B	CDKN1B
ACTG2	CDCA3	AKT3	PLN	SETD2	CDKN1B	MAPK14	DMD
ATRX	CDCA8	AMPH	NKX2-1	TNNC1	ESRRA	PLN	PLN
CFL2	CENPF	ANXA2	MITF	SLC25A4	MAPK14	VASP	NKX2-1
CKM	CKAP2	ANXA4	TMEM47	CAP1	DMD	KCNH2	TMEM47
CORO1C	DLGAP5	APBB2	KCNMB1	ANXA6	SLC35A2	MITF	KCNMB1
CSRP1	FEN1	APITD1///PEX14	COL1A2	STX10	SRP54	TMEM47	COL1A2
DAPK3	FOXM1	ARHGEF9	SLC2A4	CD248	VASP	TRDN	TRDN
DEFB129	GMNN	ATP2B1	MYL6	ACTR3	TMEM47	SLC2A4	PPARG
DIXDC1	HMGB2	ATP2B4	ACTA1	TAZ	MCL1	SGK1	IGF1
DUSP2	HMMR	ATP2C1	MYL9	ASB2	KCNMB1	NRAS	FGF8
DUSP5	KIF18B	ATP5S	DSTN	IER2	SGK1	PPARG	MYL6
EGR1	KIF20A	ATRN	LOC100686101///PLCB3///PRDX5	ACTG2	MMP14	FGF8	ACTA1
EGR2	KIF2C	ATRX	MYL12A	MYH11	PPARG	MYL6	MYL9
EPHB1	KIF4A	ATXN1	CDC14A	ITGB1BP2	FGF8	ACTA1	LOC100686101///PLCB3///PRDX5
FGFRL1	LOC481325///	BCKDHB	ITGA7	VCL	MYL6	MYL9	ATF4
FLNA	MCM4	BDNF	MYL7	THBS1	ACTA1	DSTN	ITGA7
HOXC5	MELK	BHLHE40	SETD2	MUS81	MYL9	LOC100686101///PLCB3///PRDX5	MYL7
HOXD10	NCAPH	BMPR1A	TNNC1	FOS	TPM1	ATF4	SETD2
INSM1	NDC80	CACNA1A	MYO1B	CFL1	DSTN	EEF1B2	TNNC1
KCTD15	NUSAP1	CAP2	ACTA1///ACTC1	FLNA	LOC100686101///PLCB3///PRDX5	MYLPF	ACAA2
KLF6	PCNA	CAV1	SLC25A4	ACTN1	TMOD1	MYL7	ACTA1///ACTC1
LIN37	PLK4	CDC42BPA	CAP1	SUSD1	EEF1B2	ACTA1///ACTC1	NPM3
LOC100686101///PLCB3///PRDX5	PRC1	CDK13	LMCD1	CFL1///LOC609010	MYLPF	SLC25A4	SLC25A4
LOC481325///UBE2C	PTTG1	CDKN1B	MAP1A	SCOC	MYL7	TIMP3	ZEB1

LSP1	RACGAP1	CDON	RSU1	PLCB3///PRDX5	TNNC1	MAP1A	SEPX1
MAPKAPK2	RFC4	CELF2	LCP1	TPM1///TPM3	MYO1B	RSU1	PRELP
MBNL1	RRM1	CITED2	STAT5B	MYL6///MYL6B	ACTA1///ACTC1	LCP1	MAP1A
MRGPRF	RRM2	COL1A1	TAGLN	GPR20	NPM3	STAT5B	KIF1B
MYH11	SHCBP1	COL1A2	FHL2	EGR1	SLC25A4	TAGLN	LDHA
MYL7	SMC4	COL3A1	ZNF513	PHOX2B	MAP1A	MYADM	RSU1
MYLK	SPAG5	COL5A2	ANXA6	CNN2	RSU1	ATF3	STAT5B
NKX2-1	TOP2A	CYR61	HNF1B	CA3	STAT5B	ZNF513	TAGLN
NKX2-2	TPX2	DAB2	AARSD1///BECN1	EGR3	TAGLN	ANXA6	MYADM
NPAS2	TYMS	DBP	STX10	NPAS4	ZNF513	AARSD1///BECN1	TES
NPM3	UBE2C	DDAH1	CD248	EGR2	ANXA6	STX10	ZNF513
NPPA	UBE2S	DLC1	ACTR3	FOSL1	AARSD1///BECN1	CD248	ANXA6
NR4A1	WHSC1	DLG1	TAZ	DIXDC1	STX10	ACTR3	AARSD1///BECN1
PCDH7	ZWINT	DUSP1	ASB2	HOXB4	NPPA	NPPA	STX10
PDLIM5		DYRK1A	IER2	TGFB1I1	DDX17	PPAP2B	CD248
PHF12		EFEMP1	SYT9	IL17B	PPAP2B	AKIRIN2	ACTR3
PICALM		ERBB2	ACTG2		SORT1	TAZ	NPPA
PLCB3///PRDX5		F3	SSH3		AKIRIN2	ASB2	PPAP2B
PLN		FAM179B	LYRM1		ARHGEF17	TIMP3	AKIRIN2
PODN		FBLN5	SPAG7		TAZ	IER2	TAZ
PPP1R12A		FHL2	BCAS3		TAZ	ACTG2	FST
PRDM1		FYN	LPP		FST	TPM2	PITPNA
PSENEN		FZD2	GFPT2		SCUBE3	LPP	ACTG2
PTCH1		GCNT1	RBBP7		IER2	RBBP7	ASPH
RAB30		GJA1	MATR3		ACTG2	LRRFIP1	LYRM1
RARB		GRK5	DUSP2		ASPH	MYH11	TPM2
RASSF2		ICA1	MYH11		LYRM1	ITGB1BP2	LPP
RUNDC1		ID1	DNAJB4		TPM2	CHD2	RBBP7
SCN3B		IGF1R	ACVR1		KLHL3	G3BP2	MATR3
SCOC		IGFBP5	ITGB1BP2		LPP	RARB	EPB41L4B
SREK1		INPP4B	AKAP12		RBBP7	RAI2	DUSP2
SRF		INSIG1	CYR61		DUSP2	BLOC1S1	LRRFIP1
SUSD1		IRS1	G3BP2		MYH11	SERTAD4	MYH11
SVIL		ITGB3	RRM1		ITGB1BP2	EML4	ITGB1BP2
SYT9		KALRN	DAPK3		AKAP12	CORO1C	G3BP2
TAGLN		KIT	DMD		PLK1S1	VCL	RRM1
TCF4		LAMC1	EML4		CHD2	TANK	RARB
TFE3		LDLR	MCAM		G3BP2	NR2F2	RAI2
THBS1		LOC100686073///MT1E	VCL		RARB	THBS1	BLOC1S1
TMEM47		LPAR1	TANK		BLOC1S1	PDLIM5	EML4
TNNC1		LPHN2	NR2F2		NCOA6	KDM3A	CORO1C
TPM2		LTBP1	THBS1		SERTAD4	SRF	VCL
TRDN		MAGI2	PDLIM5		EML4	MUS81	NR2F2
U2AF1L4		MAP1B	SRF		VCL	HOXC5	THBS1
UBE2C		MAP2K5	SYNCRIP		TANK	FOS	PDLIM5
ZAK		MAPK14	MUS81		NR2F2	CFL1	KDM3A
ZNF644		MET	FOS		THBS1	MBNL1	SRF
		MGLL	RAB30		PDLIM5	FLNA	SYNCRIP
		MGMT	CFL1		KDM3A	CFL2	WNT5A
		MIOS	MBNL1		SND1	ZAK	MUS81
		MMP16	FLNA		SRF	TADA3	HOXC5
		MRPS31	CFL2		MEIS2	PICALM	FOS

MT1E	ZAK	MUS81	NRXN3	CFL1
MTA1	TADA3	HOXC5	MYLK	PDLIM3
MYC	LRP5	FOS	ACTN1	MBNL1
NEK7	TUFT1	RAB30	MYO1E	FLNA
NFIB	LTBP1	CFL1	PPP2R3A	CFL2
NFKB1	MYLK	MBNL1	SUSD1	ZAK
NIPBL	ACTN1	FLNA	SVIL	TADA3
NOTCH2	MYO1E	CFL2	GPBP1	LRP5
NR1D2	PPP2R3A	ZAK	MAPKAPK2	PICALM
NR3C1	SUSD1	TADA3	ASPA	AGL
NRP1	HERC1	MYLK	CSRP1	MYLK
PAPD7	ASPA	ACTN1	CFL1///LOC609010	ACTN1
PDGFRB	CFL1///LOC609010	MYO1E	SCOC	MYO1E
PDLIM5	SCOC	PPP2R3A	ARPC4	PPP2R3A
PHF3	ARPC4	SVIL	FLNC	SUSD1
PIAS3	LDB3	HERC1	CNN1	SVIL
PIK3CD	CNN1	MAPKAPK2	THBS1	HERC1
PLCB4	DMPK	ASPA	KLF6	ATP1A2
PMP22	KLF6	CSRP1	MYADM	MAPKAPK2
PPAP2B	TOP1	CFL1///LOC609010	LOC100855913///MYADM	CFL1///LOC609010
PPARG	MYO18B	SCOC	TMEM126A	SCOC
PRDM2	PDLIM7	ARPC4	PDLIM7	ARPC4
PRKAR2B	PDLIM4	CNN1	ABR	AQP2
PRKCA	POPDC2	KLF6	SUN2	LDB3
PRKCE	ZEB2	CHMP2B	SRF	CNN1
PTEN	SUN2	ESRRA///KCNK4	MAPKAPK2	DMPK
PTGFR	SRF	SERPINH1	LIX1L	KLF6
PTPN21	SNX27///TUFT1	ABR	MAP2K6	MYADM
PTPRM	SLC7A1	STAG2	BNC2	LOC100855913///MYADM
RASA2	MEIS1	TBL1X	BNC2	MYO18B
RBPMS	DUSP6	ATRX	PLCB3///PRDX5	TMEM126A
RGS4	MYL6	MAP2K6	LDB1	PDLIM7
RND3	PLCB3///PRDX5	DUSP6	MED13	CABP1
RUNX1	MYL12A	BNC2	CSRP1	PDLIM4
RXRA	KALRN	PLCB3///PRDX5	HOXC4	RAP1A
SCAF8	AAK1	MED13	MRGPRF	ABR
SCHIP1	LRP5	MRGPRF	ZNF238	SUN2
SDC2	MRGPRF	HOXA3	TPM1///TPM3	SRF
SERPINE1	HOXA3	TPM1///TPM3	CRH	LSP1
SFMBT1	TPM1///TPM3	CRH	GPR162	SLC7A1
SIPA1L1	COL8A1	MYL6///MYL6B	MYL6///MYL6B	MAP2K6
SLC22A18	MYL6///MYL6B	COL23A1	COL23A1	CHD6

SLC7A1	*PAN2*		*CITED2*	*CITED2*	*ADAMTS12*
SMAD2///SMAD3	*LOC100682672///LOC100855903///YWHAZ*		*GPR20*	*GPR20*	*EPHB1*
SMAD3	*GPR20*		*EGR1*	*EGR1*	*PLCB3///PRDX5*
SNAI2	*EGR1*		*PTCH1*	*PTCH1*	*CNTF*
SPOP	*PTCH1*		*ADAMTSL1///LOC100686662*	*UBE2H*	*LRP5*
SRI	*RASD2*		*RASD2*	*UBE2H*	*MRGPRF*
SYNE1	*SRF*		*NUAK1*	*ADAMTSL1///LOC100686662*	*HOXA3*
SYNJ2	*GADD45G*		*GADD45G*	*GADD45G*	*TPM1///TPM3*
TFPI	*NPAS2*		*NPAS2*	*NPAS2*	*MYL9*
TGFBR2	*MYL12A///MYL12B*		*HOXA5*	*MLIP*	*MYL6///MYL6B*
TGFBR3	*HOXA5*		*FOXP2*	*ITGB8*	*CITED2*
TJP1	*LOC481684///RGS3*		*ROCK2*	*ABCA1*	*GPR20*
VAV2	*RGS3*		*ELAVL4*	*OTX1*	*EGR1*
VLDLR	*FOXP2*		*DLL1*	*FOXP2*	*PTCH1*
WDR37	*PNKP*		*KBTBD12*	*FAM131B*	*FAM83H*
YTHDC1	*ROCK2*		*CKM*	*SCOC*	*CSNK1E*
ZMIZ1	*SCOC*		*GAB2///NARS2*	*ELAVL4*	*RASD2*
	ELAVL4		*GAB2///NARS2*	*DLL1*	*SRF*
	DLL1		*RSF1*	*CKM*	*LDHA///LOC485449///LOC490690*
	DACT3		*NKX2-2*	*TMEM126A*	*GADD45G*
	CKM		*INSM1*	*RSF1*	*NPAS2*
	RSF1		*PPP1R12A*	*NKX2-2*	*HOXA1*
	NKX2-2		*RASSF2*	*PPP1R12A*	*HOXA5*
	PPP1R12A		*FOXP1*	*CNN2*	*FOXP2*
	CNN2		*NR2F1*	*RASSF2*	*ROCK2*
	RASSF2		*LRRTM4*	*CDC25B*	*PRDM1*
	STARD13		*ACTA1///ACTC1*	*STARD13*	*SCOC*
	FOXP1		*MEIS2*	*TRIM3*	*ELAVL4*
	DEFB129		*CX3CL1*	*FOXP1*	*KBTBD12*
	TRIM55		*EGR3*	*CTNND1*	*GPR158*
	KCTD15		*TANK*	*CTNND1///ZDHHC5*	*GNG8*
	PATL1		*DUSP5*	*CTNND1///ZDHHC5*	*CKM*
	CTNND1		*IP6K1*	*NR2F1*	*TMEM126A*
	CTNND1///ZDHHC5		*TBL1X*	*LRRTM4*	*RSF1*
	CTNND1///ZDHHC5		*MID1*	*ACTA1///ACTC1*	*NKX2-2*
	NR2F1		*TGIF1*	*CA3*	*INSM1*

ACTA1///ACTC1	*TAF5L*	*EGR3*	*PPP1R12A*
CA3	*ACTA1///ACTA2*	*ITGB6*	*CNN2*
ARHGAP1	*DVL3*	*TANK*	*RASSF2*
EGR3	*NPAS4*	*DUSP5*	*STARD13*
TSPAN2	*MAP1A*	*SLC16A6*	*FOXP1*
TSPAN2	*GRHL3*	*CORO1C*	*TRIM55*
ITGB6	*EGR2*	*LIX1L*	*NR2F1*
DUSP5	*HOXD10*	*MID1*	*LRRTM4*
LRP5	*SHF*	*TAF5L*	*ACTA1///ACTC1*
MCAM	*MYL1*	*ACTA1///ACTA2*	*AQP2///AQP5*
MYLK	*XK*	*DVL3*	*DHH*
RNF39	*VGF*	*ATF3*	*CX3CL1*
ACTA1///ACTA2	*DIXDC1*	*NFATC4*	*NNAT*
ACTA1	*RUNDC1*	*NPAS4*	*EGR3*
DVL3	*ACTB///ACTBL2///ACTG1*	*CD248*	*ACTB///ACTBL2///ACTG1*
OSM	*VCL*	*EGR2*	*BDNF*
LIN28A	*STAT5B*	*CFL2*	*NAV1*
NPAS4	*STAT5B*	*WNT3*	*DUSP5*
CD248	*MSX1*	*HOXD10*	*LRP5*
MAP1A	*SLC4A3*	*DUSP10*	*SCN3B*
EGR2	*RHOJ*	*SHF*	*ACTA1///ACTA2*
FOSL1	*RHOJ*	*XK*	*ACTA1*
SIPA1	*PADI2*	*DIXDC1*	*DVL3*
HOXD10	*KRT17*	*DIXDC1*	*NFATC4*
DUSP10	*GRK6*	*ARF6*	*NPAS4*
XK	*NPPA*	*RUNDC1*	*EGR2*
DIXDC1	*FGFRL1*	*PELI2*	*FOSL1*
RUNDC1	*HOXB4*	*STAT5B*	*CFL2*
RHOJ	*TGFB1I1*	*SLC4A3*	*SIPA1*
RHOJ	*NLGN3*	*RHOJ*	*HOXD10*
PADI2	*TMEM92*	*PADI2*	*DUSP10*
PDLIM7	*TRIM46*	*PDLIM7*	*DMD*
GRK6	*KRTCAP2*	*PCDH7*	*MYL1*
FGFRL1	*TPM1///TPM3*	*GRK6*	*DIXDC1*
HOXB4	*CNN1*	*NPPA*	*DIXDC1*
TGFB1I1	*POU3F4*	*FGFRL1*	*RUNDC1*
FBXL22	*TLE3*	*HOXB4*	*MSX1*
FOS	*DCUN1D3*	*TGFB1I1*	*SLC4A3*
TRIM46	*IL17B*	*FOS*	*RHOJ*
KRTCAP2	*EMILIN2///SMCHD1*	*TRIM46*	*PADI2*
TPM1///TPM3	*STAG2*	*KRTCAP2*	*RAB27A*
TPM1///TPM3	*STAG2*	*KIAA1737*	*FOXP3*
CNN1	*ELF4*	*NRXN3*	*KRT17*
POU3F4	*PHF12*	*TPM1///TPM3*	*PCDH7*
TLE3	*PODN*	*CNN1*	*GRK6*
ASB2	*CACNA1B*	*TLE3*	*FGFRL1*
LYRM1	*RERE*	*ASB2*	*FGFRL1*
DCUN1D3	*TMEM56*	*ASB2*	*ORAI3*
HNF1B	*ZNF644*	*IL17B*	*HOXB4*

UNC45B	*AGRP*	*EMILIN2///SMCHD1*	*TGFB1I1*
IL17B		*PHF12*	*FBXL22*
EMILIN2///SMCHD1		*PHF12*	*TRIM46*
PHF12		*PODN*	*KRTCAP2*
CRK		*ASPA*	*TPM1///TPM3*
WDR81		*CACNA1B*	*TPM1///TPM3*
WDR81		*TAZ*	*CNN1*
CACNA1B		*RERE*	*POU3F4*
TAZ		*ZNF644*	*TLE3*
TAZ		*NOB1*	*DCUN1D3*
CNN2			*SIN3A*
ABL1			*IL17B*
ZNF644			*EMILIN2///SMCHD1*
			PHF12
			PHF12
			PODN
			CACNA1B
			SEPX1
			TAZ
			RERE
			KIF1B
			ABL1
			ZNF644

Table F. List of genes used to perform hierarchical clustering to classify genes as basal or luminal subtypes of breast cancer and genes participating in *P53* pathways.

Basal	Luminal	*P53 RB* Pathway Genes
MMP9	*PPARG*	*NOXA1*
MMP2	*TJP3*	*BAX*
LAMA3	*FUCA1*	*HDAC1*
TCN2	*PPL*	*PDK1*
ALDH1A1	*TRIM28*	*CDC25C*
MITF	*CXADR*	*E2F1*
MYC	*TBX2*	*APC*
TRIM36	*ERBB2*	*PERP*
TSPAN7	*ERBB3*	*PML*
RNASE1	*SULT1A1*	*PDK2*
ACOT7	*MAL*	*CCNE2*
TPPP3	*TJP2*	*MDM2*
CCL8	*STXBP2*	*E2F3*
SOX9	*TGM1*	*CCND1*
RAC1	*VHL*	*PTEN*
TIMP2	*TMEM97*	*PDK1*
SERPINA3	*BID*	*CTNNB1*
MSN	*FBP1*	*MYC*
cd44	*CAPN5*	*PDK2*
AHNAK2	*RNF128*	*PDK3*
JUN	*UPK2*	
ALOX5AP	*UPK1A*	
EMP3	*CYP4B1*	
GLIPR1	*TMPRSS2*	
PALLD	*PLEKHG6*	
PRKCDBP	*VGLL1*	
PRRX1	*FGFR3*	
ZEB1	*KRT8*	
ZEB2	*KRT7*	
VIM	*CYP4B1*	
EGFR		
MCL1		
STAT1		
STAT3		
RAC2		
SERPINA5		
STAT4		
